# Grasp and Release Test for Tetraplegic Hand Assessment: an update of the Grasp and Release Test

**DOI:** 10.1038/s41393-023-00907-0

**Published:** 2023-07-04

**Authors:** Jennifer A. Dunn, Johanna Wangdell, Anne Bryden

**Affiliations:** 1 Department of Orthopaedic Surgery and Musculoskeletal Medicine, University of Otago, Christchurch, New Zealand; 2 Centre for Advanced Reconstruction of Extremities, Sahlgrenska University Hospital/Mölndal, Mölndal, Sweden; 3 Department of Hand Surgery, Institute of Clinical Sciences, University of Gothenburg, Gothenburg, Sweden; 4 MetroHealth Department of Physical Medicine and Rehabilitation, MetroHealth Center for Rehabilitation Research, Cleveland, OH USA; 5 Institute for Functional Restoration, Case Western Reserve University, Cleveland, OH USA

**Keywords:** Outcomes research, Rehabilitation

## Abstract

The Grasp and Release Test (GRT) was originally developed to measure effectiveness of an implanted neuroprosthesis in people with tetraplegia. Its ease of use and lack of floor and ceiling effects culminated in recommendations for inclusion in a battery of tests to measure outcome following upper limb reconstructive surgery. However, the length of time taken to administer the GRT in a clinical setting, lack of instructions of accepted grasp patterns in the upper limb reconstructive surgery population and scoring procedures lead to differences in reporting outcomes using this measure. In order to ensure clinical utility for the upper limb reconstructive surgery population, revisions of the original test instructions have been made and are reported in this article. Further testing of the psychometric properties of the new measure are currently underway.

## Introduction

Recommendations of outcome measures to assess upper limb reconstructive surgery interventions such as tendon transfer and/or nerve transfer procedures in individuals with tetraplegia have been well documented [1, 2]. The Grasp and Release Test (GRT) has been recommended for inclusion in the battery of tests for this population by an International Upper Limb Surgery Therapist Consensus group since 2007 [3].

The GRT is a non-instrumented, one-handed object manipulation test used to measure hand function in people with tetraplegia. The GRT was originally designed to measure effectiveness of an implanted neuroprosthesis [4, 5]. The psychometric properties of the GRT were established by the developers for use with an implanted neuroprosthesis [5] and later by Mulcahey et al. in people with tetraplegia undergoing tendon transfer surgery [6]. The GRT demonstrated good test-retest reliability and demonstrated the ability to detect changes in hand function before and after tendon transfer surgery [6]. The GRT has been used to measure changes in hand function as a result of upper extremity interventions in tetraplegia, such as tendon and/or nerve transfers [7, 8] and tendon lengthening due to upper limb spasticity in persons with tetraplegia [9]. It has also demonstrated a high degree of responsiveness to changes in function during rehabilitation in persons with tetraplegia [10].

Until recently, GRT use has mostly been limited to centres previously involved in a multi-site trial to evaluate an implanted neuroprosthesis for hand function [4]. Due to its clinical utility for assessing impacts of upper limb reconstructive surgery and increased interest in the test by other tetraplegic hand surgery centres, the GRT has been made commercially available (https://neuraloutcomes.com/product/grasp-and-release-test/). While the GRT has been used to report changes in grasp and release following upper limb reconstructive surgery [7, 8], utility of the measure in a clinical setting was questioned due to its administration time and anecdotal redundancy of the required pre-test and multiple trials per object for the reconstructive surgery population. The original GRT pre-test was devised to determine objects that the individual was able to successfully grasp and release for inclusion in the main test, and identify whether grasp parameters of the neuroprosthesis were appropriate [5]. If requirements for parameter changes were identified during the pre-test, the test was concluded and repeated after grasp modifications were made. Anecdotally, for the reconstructive surgery population it was felt that a formal pre-test was not required as grasp modifications were not an option and a simple ‘practice’ of the item for inclusion in the main test would be sufficient. Additionally, the GRT required three timed trials of each object in the main test, however clinical experience in the upper limb reconstructive surgery population demonstrated redundancy in administration as very little change was observed in the number of completions across the three trials. Further, specific scoring instructions for the GRT have never been published, resulting in variability in published results. Wuolle et al. reported median completions and failures across five trials [5]. Mulcahey et al. reported the mean number of completions for each of the objects separately for the three trials as well as how many of the six objects were able to be grasped and released before and after surgery [6]. Peckham et al. also reported how many of the six objects participants were able to be grasped and released with and without the neuroprosthesis [4]. In contrast, van Zyl et al. [7] and Wangdell et al. [8, 9] reported a single mean total of completions for all objects and all trials noting that an increase in mean total score was due to improvements in the number of items able successfully grasped and released by the participant and/or less failures. To further increase clinical utility and standardisation of the GRT for measuring surgical outcomes, minor revisions of the testing procedure and standardised scoring instructions were needed. Therefore, the GRT for Tetraplegic Hand Assessment (GRT THA) was developed. Six centres (based in United States of America, Sweden, Australia, New Zealand, Switzerland and Netherlands) specialised in interventions for the tetraplegic upper limb were involved in the development of the revised testing procedure.

## Grasp and Release Test

**Fig. 1:**
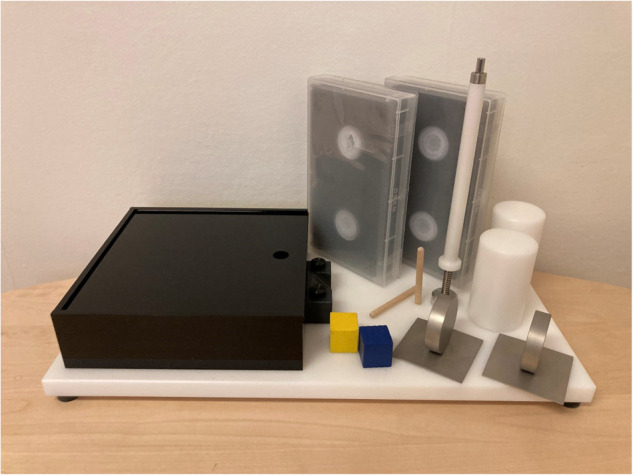
Grasp and Release Test.

The GRT includes six objects that participants are asked to grasp, move and release as many times as possible in 30 s. The six objects tested in the GRT are; peg, paperweight, fork, block, juice can and videotape/book (Fig. 1).

The objects vary in size, weight and surface texture. The objects represent a range of difficulties that can measure differences in performance. The test has been designed to minimise effects of extraneous factors which may interfere with the performance of the hand, such as fatigue of the proximal musculature or instability of the trunk. Therefore, the test board is placed in front of the participant and biased towards the test hand as needed to minimise reaching effort. The test stipulates consistent equipment placement and test board location so that performance over time and across participants may be compared meaningfully. The objectives of the GRT are: (1) to determine if ability to acquire, grasp, move and release a series of objects ranging in size and weight changes; and (2) to determine ability to acquire, grasp, move and release objects changes as a result of an intervention or changes over time.

The original test to assess neuroprosthetic hand function in a research setting consisted of a pre-test to determine success or failure in acquiring, moving and releasing the object (including practice), followed by a main test with three 30-s trials for each successful object in the pre-test. The number of completions and failures in the 30-s trials are recorded, with comparisons made between objects successfully acquired with the neuroprosthesis turned on and off (maximum of six). Changes were made to the GRT testing procedure to standardise administration and maximise clinical efficiency in measuring impacts of upper limb reconstructive surgeries.

## Changes to testing procedures

Fundamentally the GRT THA remains very similar to the original GRT designed by Woulle et al. [5]. The changes were developed due to the differences in testing the hand following implantation of a neuroprosthesis compared to following upper limb reconstructive surgery. Specific changes to the testing procedures are:

1. Clarification of accepted prehension patterns taking into account the variability of grasps produced by upper limb reconstructive surgery.
2. Removal of specific pre-test but allowing practice of each object prior to testing.
3. Elimination of random testing order for objects, and change to tasks being performed in the order of the scoring sheet.
4. Reduction of testing trials for each object from three times down to once.
5. Standardisation of fork testing position to only allow vertical position for testing.
6. Only recording number of successful completions of objects within the 30 s trial.
7. Scoring–sum score of successfully moved items across all six tasks.

The changes to the testing procedures reduce time and effort for administering the test in a clinical environment. Due to the variability of grasps created by upper limb reconstructive surgeries, definitions of accepted grasp types and adaptations for the GRT THA were established. The original GRT specified three objects to be acquired using lateral grasp (peg, paperweight, fork) and three using palmar grasp (block, can, videotape). The GRT THA allows object acquisition with any surgically provided grasp pattern, within specific documented instructions. Changes to grasp patterns include participants allowed to pick up the peg, videotape/book and paperweight with the thumb and another finger (as opposed to a key pinch between the thumb and index finger as required in the GRT). For the block, accepted grasp patterns include both a gross grasp of the block between all fingers and the palm of the hand (as in a tenodesis action) or between thumb and index finger. For the can, the grasp pattern must use at least three fingers and the cylinder must be grasped by the sides with the top exposed (mimicking a grasp required for drinking).

The original GRT pre-test was considered important to allow for adjustment of neuroprosthesis parameters and practice in its use [5], therefore its removal from the GRT THA is feasible as no parameters are able to be adjusted post upper limb reconstructive surgery. Additionally, the original GRT measured consistency of neuroprosthesis performance over time by requiring three trials per object and random-order testing to minimise errors due to learning or fatigue [5]. However, for individuals undergoing upper limb reconstructive surgery, eliminating the pre-test and reducing the number of trials in the GRT THA minimises risk of fatigue. Finally, in the original GRT the number of completions and failures for each object was recorded. Again, the recording of failures was to evaluate the consistency of the performance of the neuroprosthesis over time. In the upper limb reconstructive surgery population, consistency of performance is not such an issue and as such failures are not recorded. The instruction manual for the GRT THA is currently being finalised and instructions for download will be available free of charge at (https://neuraloutcomes.com/product/grasp-and-release-test/) once finalised. It is not expected that there would be a need for training for clinicians using the GRT THA as comprehensive instructions are provided in the instruction manual. The GRT THA testing kit can be purchased from this website and has a current cost of US$2500.

## Recommendations for use

Within the recommended battery of tests following upper limb reconstruction in people with tetraplegia, the GRT is currently the only standardised physical performance activity test with all others being patient-reported outcomes [1]. The GRT THA procedure has been developed specifically for the tetraplegic population undergoing upper limb reconstructive surgery procedures. The objects tested within the GRT THA are of similar size and weight to replicate many everyday items used by the tetraplegic population and the prehension patterns (pinch and grasp) are the two most commonly reconstructed grip patterns. This reduces redundancy of testing in this population. The advantages of using the GRT THA compared to the GRT are reduced time for test completion, simplified testing and scoring, and standardisation of grip patterns available following upper limb reconstructive surgery. While the time taken to administer the GRT THA is dependent on the number of objects the individual is able to grasp and release, it is expected that the time to administer the test will be reduced by at least a third (for example to administer the GRT in the upper limb reconstructive surgery population with six objects takes up to 60 min, compared to the GRT THA with six objects which takes between 15–20 min). The disadvantage of using the GRT THA is that currently there are no psychometric data available for the GRT THA.

## Conclusions

The commercial availability of the GRT and the revised GRT THA testing procedure provides opportunity for all centres currently performing upper limb reconstructive surgery procedures to be able to quantify changes in hand function. Increased uptake of such outcome measure use facilitates the ability to combine and compare results from all centres and provide meaningful information to health professionals, people with tetraplegia, and funders regarding the effectiveness of such procedures. The psychometric properties of the GRT THA are currently being investigated.
